# Navigating the Challenges: Construction Lessons in Establishing an Orthopedic Institute in a Sterile Environment

**DOI:** 10.1017/ash.2024.227

**Published:** 2024-09-16

**Authors:** Ruby Mathew, Mona Shah

**Affiliations:** Endeavor Health - Northshore University Health System; NorthShore University HealthSystem

## Abstract

**Background:** In order to become a new, state-of-the-art orthopedic institute with additional operating rooms, and a brand new sterile processing department (SPD), construction needed to be done within a sterile environment. Expansion of the operating room (OR) included adding a whole new wing, with smaller construction work within the open and active OR. While construction for a new SPD occurred in the midst of an active and open SPD; unlike the addition of the OR, new SPD construction included heavy demolition and multiple dust generating projects. There were many lessons learned from the built of this brand new institute, which took place in the middle of a pandemic era. **Method:** Each project involved in the making of the new orthopedic institute required an Infection Control Risk Assessment (ICRA), as well as walkthroughs and team meetings prior to the start of work. Team meetings involved the hospital Infection Preventionist (IP), the construction project manager, and the departments [involved] managers. Requirements, to mitigate dust dispersal into the neighboring sterile environment, were listed in the ICRA. In addition, weekly rounding was conducted by the team to ensure air flow requirements were followed, and there were no accumulation of dust dispersing to the sterile side. Reprocessing was also conducted at sister facilities when existing reprocessing items (sterilizers, instrument washers, cart washers, etc) were shut down; the hospital also went on bypass a few times in order to accommodate patients who emergently needed operating services. **Result:** By April 2022, after two years of construction, the new orthopedic institute with 12 new operating rooms, new ambulatory surgery units (ASU), new post anesthesia care units (PACU), and a brand new SPD went live. There were more than 100 shutdown notices, over 50 alternative practices in place to continue daily operations while also allowing construction work to continue. Noncompliance to any of the requirements was immediately followed up with an urgent notice to the project manager. **Conclusion:** Hindsight, heavy construction in a sterile environment is not preferred and it would have been easier to add on a new SPD, similar to how the OR additions were added; instead of building within an actively open sterile environment. Multidisciplinary team meetings conducted at the very beginning of the project would have prevented many shut downs and alternate practices; . It is pertinent that IPs and clinical department managers are involved at the most earliest phases of the construction.

